# Twadn: an efficient alignment algorithm based on time warping for pairwise dynamic networks

**DOI:** 10.1186/s12859-020-03672-6

**Published:** 2020-09-17

**Authors:** Yuanke Zhong, Jing Li, Junhao He, Yiqun Gao, Jie Liu, Jingru Wang, Xuequn Shang, Jialu Hu

**Affiliations:** 1 School of Computer Science, Northwestern Polytechnical University, West Youyi Road 127, Xi’an, 710072 China; 2 Xi’an Mingde Institute of Technology, Fenghe Campus, Fenghe Campus, Xi’an, 710124 China; 3 Centre of Multidisciplinary Convergence Computing, School of Computer Science, Northwestern Polytechnical University, 1 Dong Xiang Road, Xi’an, 710129 China

**Keywords:** PPI network, Dynamic network, Network alignment, Dynamic time warping

## Abstract

**Background:**

Network alignment is an efficient computational framework in the prediction of protein function and phylogenetic relationships in systems biology. However, most of existing alignment methods focus on aligning PPIs based on static network model, which are actually dynamic in real-world systems. The dynamic characteristic of PPI networks is essential for understanding the evolution and regulation mechanism at the molecular level and there is still much room to improve the alignment quality in dynamic networks.

**Results:**

In this paper, we proposed a novel alignment algorithm, Twadn, to align dynamic PPI networks based on a strategy of time warping. We compare Twadn with the existing dynamic network alignment algorithm DynaMAGNA++ and DynaWAVE and use area under the receiver operating characteristic curve and area under the precision-recall curve as evaluation indicators. The experimental results show that Twadn is superior to DynaMAGNA++ and DynaWAVE. In addition, we use protein interaction network of Drosophila to compare Twadn and the static network alignment algorithm NetCoffee2 and experimental results show that Twadn is able to capture timing information compared to NetCoffee2.

**Conclusions:**

Twadn is a versatile and efficient alignment tool that can be applied to dynamic network. Hopefully, its application can benefit the research community in the fields of molecular function and evolution.

## Background

In recent years, due to the rapid development of biotechnology, we can obtain a large amount of biological data, such as: gene expression data, methylation data, protein interaction network data and so on [1]. Protein is a substance closely related to life and various forms of life activities. It plays a vital role in almost all life activities. Therefore, research on proteins plays a crucial role in our biological research. Protein is not a single biological function. It usually interacts with other proteins to perform certain biological functions [2–5]. All protein interactions form a protein-protein interaction (PPI) network. Most of networks are dynamic in real-world systems. For instance, PPI could change over time, and online professional network will also evolve over time [6]. A large number of PPIs are transient interactions, which briefly exists in only certain cellular context related with cell types, cell cycle stages etc. However, most of network alignment (NA) methods are designed for static networks [7], since static networks were used to model complex real-world systems. The aim of NA on PPI networks is to find an optimal node mapping that can indicate similar biological meanings between matched proteins. However, these networks actually change over time. The dynamic characteristic of PPI networks is essential for the understanding of evolution and regulation mechanism at the molecular level. Some pioneer works [8] attempt to improve NA quality using dynamic network model on evolving systems. This new computational framework can use dynamic characteristic as a supplementary information in the measure of node similarity, whereas it also suffers from the lack of high-confidence dynamic networks of real-world systems. Network study consists of a lot of parts, such as KF-finder [9], which can identify key factors from host-microbial networks in cervical cancer, besides, detection of network motif [10] is also a major search of network. In this paper, we focus on NA, which can be used to predict protein function by transferring functional knowledge from a well-studied species to a poorly-studied species.

There are two categories of alignment methods according to the target regions of networks: global alignment and local alignments. Global alignment is to find one global node mapping for compared networks [11], while local alignment aims to identify multiple conserved subregions which reflect putative functional modules of biological systems [12]. Alignments of two networks are called pairwise network alignments, those of three or more are termed as multiple network alignments. In this paper, we aim to address the global alignment problem of two dynamic networks. IsoRank was originally proposed to solve pairwise global alignment. It was intuitively guided by the assumption that one protein is a good match for another protein in the other compared network if their neighborhood topologies and sequences are similar. Many more alignment tools were developed to improve the algorithm performance of existing methods over the past decade. Among these, there are NETAL [13], H-GRAAL [14], MAGNA [15], MAGNA++ [16], which can provide one-to-one global node mapping for two compared networks. To find protein match sets for multiple species, IsoRankN [17], NetCoffee [11], SMETANA [18] and multiMAGNA++ [19] were used to find one global node mapping for multiple PPI networks. All these algorithms focused on aligning protein pairs based on static networks, although these networks evolve over time. DynaMAGNA++ [8] and DynaWAVE [20] were recently proposed to make up this deficiency. DynaMAGNA++ is the first dynamic NA algorithm, which was adapted from the MAGNA++ method. DynaMAGNA++ takes two measures (node conservation and edge conservation) to capture functionally conserved proteins. However, there is still much room to improve the alignment quality in dynamic networks. It is still a challenge to solve the alignment problem for dynamic networks.

To overcome these issues, we proposed a novel NA algorithm based on a technique termed as dynamic time warping (DTW) to align dynamic PPI networks across species. A 5-tuple-feature vector was calculated on each node of each time snapshot. A target scoring function was used to evaluate the quality of NA, which integrates both topology and sequence information. Then, the alignment problem is transformed into an optimization problem. Simulated annealing was applied to iteratively search for a near-optimal global node mapping between two compared networks.

## Methods

**Fig. 1:**
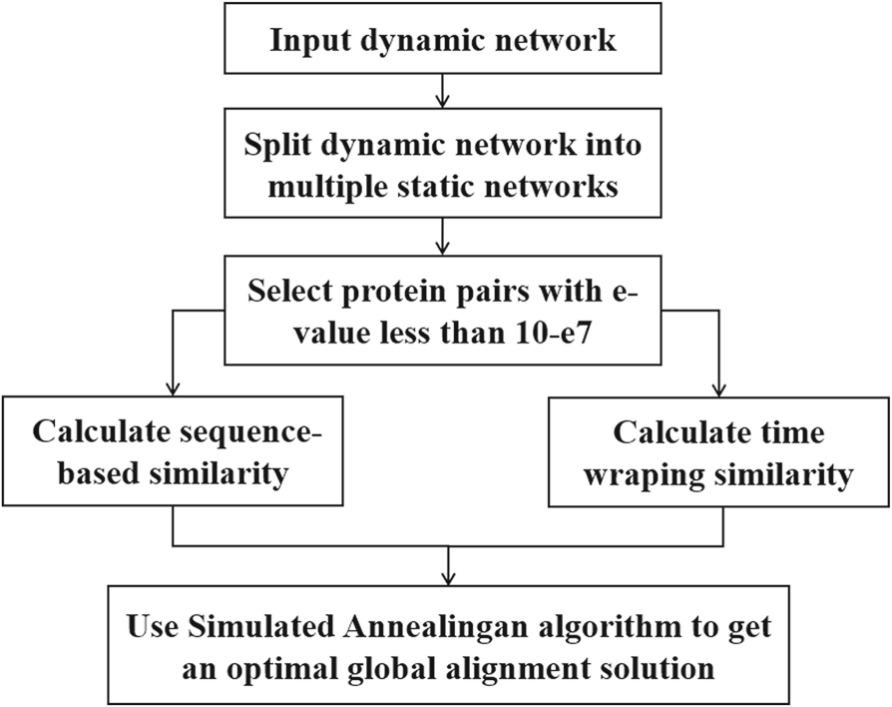
Algorithm framework of Twadn

The Twadn algorithm returns the optimal alignment results over two given dynamic networks. A dynamic network can be seen as a series of static networks based on a time sequence. So the structure feature of each static network can be extracted by a traditional static NA. In our program, one of our previous work NetCoffee2 [21] was applied to extract the topological feature of each node in the network. Then we can get a sequence of features of each vertex in the dynamic network. Simulated annealing algorithm was used to search for a near-optimal solution. Twadn’s algorithm framework is shown in Fig 1. and it has four major steps: 1) perform pair-wise sequence alignment for all pairs of proteins, and select the similar pairs which are statistically significant; 2) extract the topological feature of each vertex in each of the static network using NetCoffee2; 3) calculate the dynamic time warping similarity of all pairs of proteins; 4) use simulated annealing algorithm to find an optimal NA.

### Sequence-based similarity

We use the open source tool BLASTP [22] to sequence alignment of all proteins in the network, and obtain the sequence similarity scores e-value and bit-score for each protein pair. Considering that the amino acid sequence that affects protein function may be just a functional region of the sequence, we use the e-value parameter for preliminary filtering and select those protein pairs with e-value less than 1e-7 as the e-value can affect the coverage of predicted homologous proteins by the NetCoffee2 algorithm. Note that *Ω* denotes the candidates of homology proteins. Given a protein pair *u* and *v*, the sequence similarity *s*_*h*_(*u*,*v*) can be calculated in the following formula:

1 $$ s_{h}(u,v) = \frac{\varepsilon(u,v)-\varepsilon_{min}(u,v)}{\Delta\varepsilon}  $$

Here, *ε*(*u*,*v*) can be −*log*(*e*−*value*) or bit-score of the protein pair *u* and *v*, *Δε* is the largest difference between any two pairs of homolog in *Ω*, and *Δε*=*ε*_*max*_(*u*,*v*)−*ε*_*min*_(*u*,*v*), which servers as a normalization factor. The similarity values calculated by this method are in the interval [0, 1], where 0 represents the least similar protein pair and 1 represents the most similar protein pair.

### 5-tuple-feature vector of every vertex

Dynamic networks can be regarded as a series of static networks at many snapshots. Here, we attempt to construct a 5-tuple-feature (*γ*,*σ*,*τ*,*η*,*θ*) for each node in the static network to represent local connectivity of its corresponding node. We denote the adjacent matrix of a netwrok *G* as *M*_*n*×*n*_. Since *M* is real and symmetric, it must has a major normalized eigenvector *K*=(*k*_1_,*k*_2_,...,*k*_*n*_), which is the normalized eigenvector of the largest eigenvalue. Then, we use *k*_*i*_, 1≤*i*≤*n* as the reputation of the node *v*_*i*_ while the greater the reputation is, the more important the node is. Therefore, we use *k*_*i*_ as the first element of the 5-tuple-feature vector (i.e. *γ*) for node *v*_*i*_. The set of neighbors of *v* is denoted as *N*_*v*_. Then, we use |*N*_*v*_| as the second element of the 5-tuple-feature vector (i.e. *σ*), the sum of the reputation of these nodes ${\sum \nolimits }_{x\in N_{v}} k_{x}$ as the third element(i.e. *τ*). Let us denote nodes that are 2-step away from *v* as $N_{v}^{2}$ and all nodes in $N_{v}^{2}$ are not directly connected to *v*. Then, we use $\left |N_{v}^{2}\right |$ as the fourth element (i.e. *η*). The last element *η* is calculated by the following formula:

2 $$ \frac{1}{2} \sum\limits_{x\in N_{v}^{2}} k_{x} p_{xv}  $$

where *p*_*xv*_ presents the number of the shortest paths from *x* to *v*.

### Dynamic time warping similarity

The input for calculating the dynamic time warping similarity between networks is two dynamic network *DN*1 and *DN*2, and output is a matrix *S* in which each element *s*_*ij*_ represents the time warping similarity between protein i from *DN*1 and protein j from *DN*2. Therefore, for each protein in *DN*1, we need to calculate it’s time warp similarity with each protein in *DN*2. Here we carefully explain the calculation method of the time warping similarity between the proteins *P* and *Q* of *DN*1 and *DN*2, then through apply this method to all other protein pairs, we can obtain the result matrix *S*.

Since proteins from different dynamic network may have different snapshot number, the length of each sequence might be different. So, Euclidean distance is unable to be used in measuring the similarity of two given nodes as their differences in sequence length. Suppose *P* and *Q* appearing *m* and *n* times in their snapshots respectively, so their time sequences can be written as, *p*=(*p*_1_,*p*_2_,...,*p*_*i*_,...,*p*_*m*_),*q*=(*q*_1_,*q*_2_,...,*q*_*j*_,...,*q*_*n*_). Here, *p*_*i*_ and *q*_*j*_ are the 5-tuple-feature vectors of time sequences *p* and *q* at its *i*^*th*^ and *j*^*th*^ appearance in the snapshot.

DTW is one of the algorithms for measuring similarity between two temporal sequences, which may vary in length and it’s proved that DTW is a robust distance measure for time series, allowing similar shapes to match even if they are out of phase in the time index or different length of time series. So we apply DTW algorithm to calculate the time warping similarity between the sequences p and q.

To align two sequence using DTW, we construct a matrix *D*_*m*×*n*_ in which each matrix element value *d*_*ij*_ represents distance between *p*_*i*_ and $q_{j} \left (i.e.\ \ d_{ij}={\sum \nolimits }_{k=1}^{5} \left (p_{ik}-q_{jk}\right)^{2}\right)$, which capture the time feature of protein in the network. Here, we define a warping path to describe the time correspondence between *p* and *q* as follow:

3 $$ W=w_{1},w_{2},...,w_{k}, \max(m,n)\leq k\leq m+n-1  $$

The form of *w*_*k*_ is (*i*,*j*), which represents that this path passes through the lattice corresponding to *p*_*i*_ and *q*_*j*_. DTW is a typical optimization problem and its purpose is to find the warping path that minimizes the cumulative distance between two sequence as follow:

4 $$ DTW(p,q)=\underset{W}{\min} \sum\limits_{i=1}^{k} \delta\left(w_{i}\right)  $$

Here, *δ*(*w*_*k*_)=*d*_*ij*_ is the distance between two time series elements of *w*_*k*_.

The selection of this path needs to meet the following constraints: 1) *w*_1_=(1,1) and *w*_*k*_=(*m*,*n*); 2) If *w*_*k*−1_=(*i*,*j*) and *w*_*k*_=(*i*^′^,*j*^′^), then *i*<=*i*^′^<=*i*+1,*j*<=*j*^′^<=*j*+1. Then, the search for an optimal path can be transformed into a dynamic programming problem. We define the recurrence formula of the cumulative distance of each pair of proteins as follow:

5 $$ \lambda (i,j) = d(p_{i},q_{j}) + min \{ \lambda (i-1, j-1), \lambda (i-1, j), \lambda (i,j-1)\}  $$

where *λ*(*i*,*j*) is the dynamic time warping distance of *p* and *q*. With the warping path *W*, the node similarity of any two nodes *p* and *q* in the dynamic network can be calculated with the Gaussian function $s_{t}(p,q)=exp\left (-\frac {1}{2}{[\lambda (m, n)]}^{2}\right)$.

**Fig. 2:**
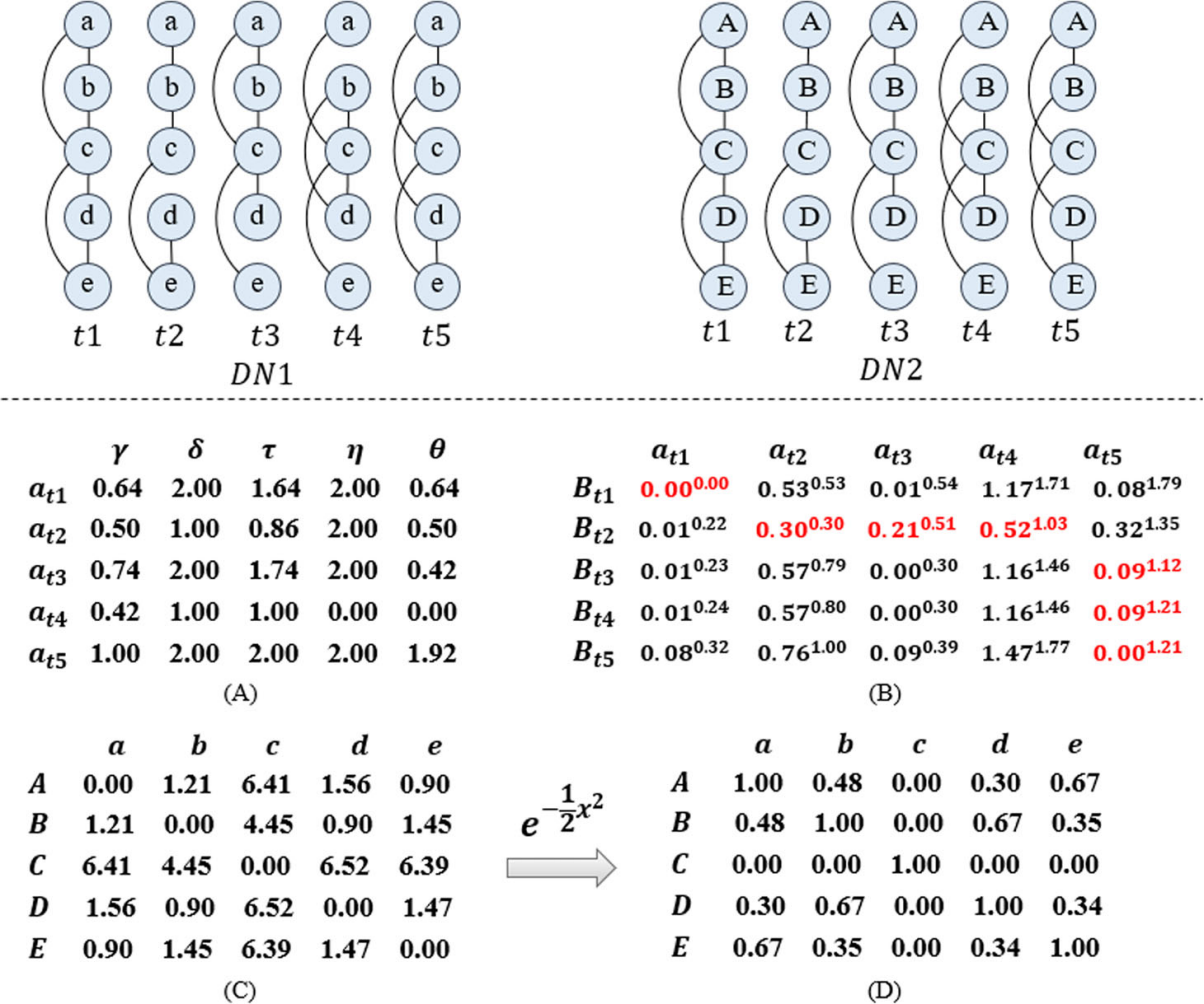
Dynamic time warping similarity between dynamic network *DN*1 and *DN*2

Figure 2 shows an example of calculate dynamic time warping similarity of each two nodes in dynamic network *DN*1 and *DN*2. The matrix (A) are the 5-tuple-feature sequence of node *a* at five snapshots. Red numbers in matrix (B) constitute the warping path, which can be used to calculate the dynamic time warping distance of node *a* and node *B*. The matrix (C) contains all dynamic time warping distance of each two nodes in *DN*1 and *DN*2. The matrix (D) contains all dynamic time warping similarity of each two nodes in *DN*1 and *DN*2.

### Simulated annealing

To find an optimal NA, Twadn used the simulated annealing technique to search for an approximately optimal solution, which maximizes the sequence similarity and dynamic time warping similarity with an objective function. The objective function writes as $f(A)={\sum \nolimits }_{m\in A} s_{m}$, where *A* and *m* refer to all possible match sets and a match set, respectively. A match set is a putative functional orthologs that could be a group of functionally related proteins. Suppose there is a match set *m*=(*m*_1_,*m*_2_,...,*m*_*v*_), the alignment score of *m* is:

6 $$ s_{m}=\sum\limits_{i,j,i\neq j} \alpha s_{h}(i,j)+\sum\limits_{i,j,i\neq j} (1-\alpha)s_{t}(i,j), i,j \in \{ m_{1},m_{2},...,m_{v} \}  $$

where *s*_*h*_(*i*,*j*) and *s*_*t*_(*i*,*j*) is sequence-based similarity and dynamic time warping similarity of protein *i* and *j* described above. With this definition, an optimal global alignment solution could be solved by maximizing a target function:

7 $$ A^{*} = arg \max \limits_A f(A) = arg \max \limits_A \sum_{m\in A} s_m  $$

## Result

To test our method, Twadn was evaluated on both simulated dynamic networks and real-world dynamic networks. We use a simulated dynamic network to compare Twadn, DynaMAGNA++ and DynaWAVE and evaluate the quality of alignment results in terms of area under the precision-recall curve(AUPR) and area under the ROC curve(AUROC). To show the ability of characterizing the time information in dynamic network, Twadn and NetCoffee2 were implemented on two real-world dynamic networks. We aggregated a dynamic network into a static network. In the static version, the network has the same set of nodes as the dynamic network, and a static edge will exist between two nodes if there is at least one edge between the same two nodes in the dynamic network. This kind of dynamic network aggregation was commonly applied in other time series network analyses, such as [23].

### Evaluation using synthetic dynamic network

#### Model of synthetic dynamic network generation

To simulate a network that mimics the evolution of protein-protein interaction networks, we generated the simulated network using a scale free gene duplication network model [20]. First, a small seed network was given with two connected nodes. Then, we add a node and some edges to network at each step, which simulates the gene duplication and divergence mechanism during the evolution of PPI networks:

**Duplication:** A node *i* is selected at random. A new node *i*^′^, with a link to all the neighbors of *i*, is created. With probability *p* a link between *i* and *i*^′^ is established.

**Divergence:** For each of the nodes *j* linked to *i* and *i*^′^, we choose randomly one of the two links (*i*,*j*) or (*i*^′^,*j*) and remove it with probability *q*.

#### Evaluation measures

A good NA approach should be able to produce high quality alignments between networks that are similar, and to produce low quality alignments between networks that are dissimilar [8]. It assumes that networks originating from a model with a same set of parameters should be more similar than these from different parameters [20]. We generate 20 dynamic networks using two sets of parameters *p*=0.3,*q*=0.7 and *p*=0.7,*q*=0.6. Each model generates 10 dynamic networks. We align all possible pairs of the synthetic networks by using Twadn, DynaMAGNA++ and DynaWAVE. Alignment quality of the $C^{2}_{20}=190$ pairs of synthetic dynamic networks can reflect the alignment power of the three algorithms. Alignment tools can classify all pairs of networks based on the alignment score into two categories, similar pairs and dissimilar pairs. With a given threshold *ρ*, these network pairs with a score *s*>*ρ* will be categorized into similar pairs, others will go to dissimilar pairs.

Afterwards, we can compare the performance of all the alignment algorithms using precision-recall and receiver operating characteristic (ROC). The precision is the fraction of network pairs which are true positive among all pairs which have a score *s*>*ρ*. The recall is the fraction of network pairs which are true positive among all pairs which are true (similar) network pairs. A precision-recall curve can be plotted by adjusting the threshold *ρ* from 0 to the maximum observed alignment score. The area under the precision-recall curve (AUPR) and F-score are two commonly used measures for the performance of classification methods. The measure F-score is the harmonic mean of precision and recall which can be calculated by following function:

8 $$ F=2\times \frac{precision \times recall}{precision+recall}  $$

We used the F-score at the point that precision and recall are equal, termed as F-score _*cross*_ and the score when F-score gets the maximum, termed as F-score _*max*_. We also evaluated the binary classification according to the receiver operating characteristic (ROC) curve, which was created by plotting the true positive rate (TPR) against the false positive rate (FPR). Here, TPR is the same as recall. FPR is the fraction of false positive network pairs among all false pairs (i.e. dissimilar network pairs). The ROC curve can be plotted by adjusting the criterion *ρ*. The area under the ROC curve (AUROC) can be calculated after we got the plot.

#### Performance on synthetic dynamic networks

**Fig. 3:**
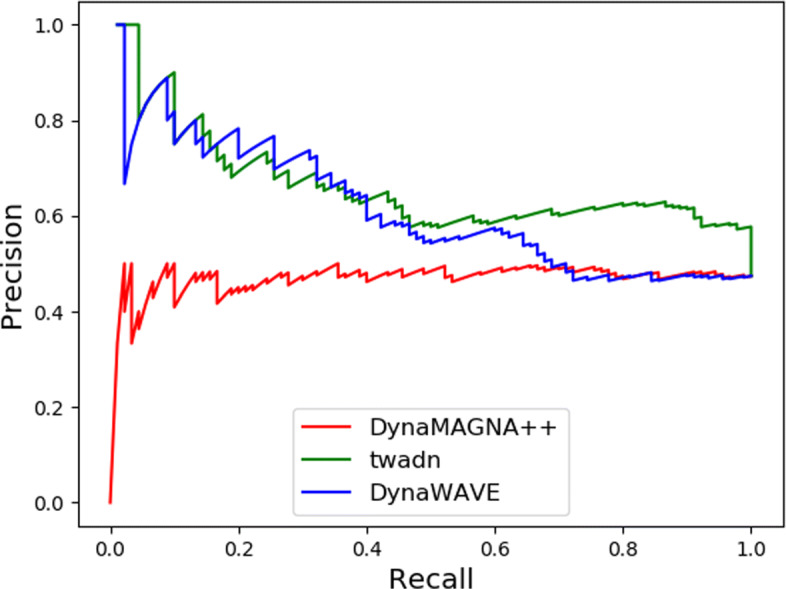
Network discrimination performance of DynaMAGNA++ and Twadn for biological synthetic networks with respect to precision-recall curve

**Fig. 4:**
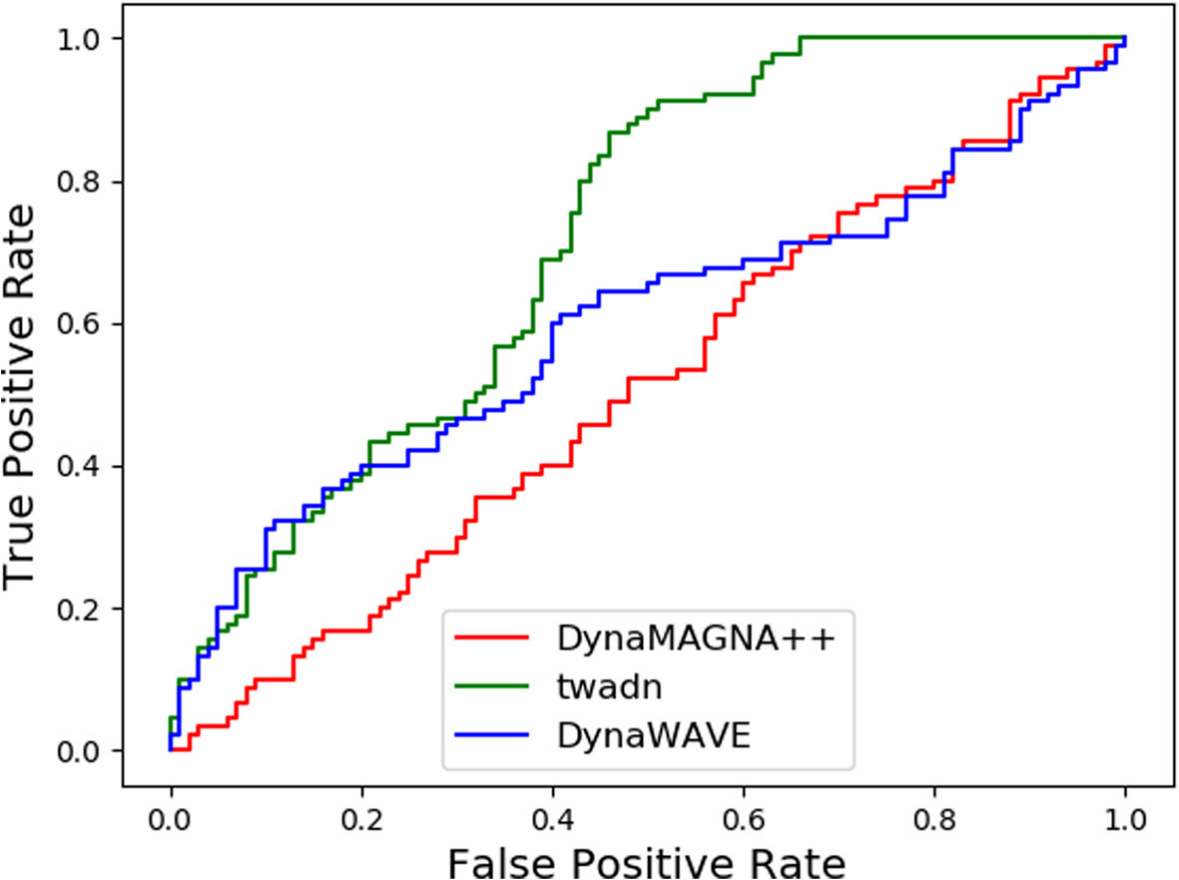
Network discrimination performance of DynaMAGNA++ and Twadn for biological synthetic networks with respect to ROC curve

For synthetic dynamic networks, we aim to develop a NA tool to distinguish these similar network pairs from the other. So we use AUPR, F-score _*cross*_, F-score _*max*_, and AUROC to evaluate the quality of the alignment algorithm. As shown in Table 1, the performance of Twadn outperforms all other alignment algorithms in terms of AUPR, F-score _*cross*_, F-score _*max*_, and AUROC, which are 0.653, 0.589, 0.735, and 0.718, respectively. DynaWAVE shows a better performance than DynaMAGNA++ in terms of AUPR, F-score _*cross*_, and AUROC. From Fig. 3, it shows that the PR curve of DynaMAGNA++ starts from origin of the coordinate system. It means that DynaMAGNA++ failed to discriminate the one with the best alignment score. From Figs. 3 and 4, Twadn is the best aligner according to both the PR curve and the ROC curve over all.

**Table 1:** Network discrimination performance of DynaMAGNA++, DynaWAVE and Twadn. For biological synthetic networks, with respect to the area under the precision-recall curve (AUPR), F-score at which precision and recall cross and are thus equal (F-score _*cross*_), maximum F-score (F-score _*max*_), and the area under the ROC curve (AUROC). In each column, the best score is bolded

algorithm	AUPR	F-score _*cross*_	F-score _*max*_	AUROC
DynaMAGNA++	0.467	0.489	0.642	0.507
DynaWAVE	0.600	0.556	0.642	0.594
Twadn	**0.653**	**0.589**	**0.735**	**0.718**

### Evaluation using real-world dynamic networks

#### Experimental design on real-world dynamic networks

To show the alignment capabilities in real-world networks, we evaluated our algorithm on PPI networks. In contrast to static NA tools, Twadn is able to capture dynamic features of a node in the time axis, which would benefit the alignment quality. It’s difficult to tell whether two biological networks come from a same evolution model or not because of the ambiguity of evolution model in biology. So we use a randomized (noisy) version of the network (see below). The larger randomized noise level, the more dissimilar the two dynamic network are. We have two randomized versions of the network. One randomizes only the temporal aspect of the network and another randomized both temporal aspect and structure aspect.

Since there is a lack of available experimental dynamic molecular networks, we create a dynamic Drosophila melanogaster PPI network from an artificial temporal sequence of static PPI networks. Here, the static PPI network that are used as snapshots of the dynamic PPI network are all real-world networks, it is just their temporal sequence that is artificial. The sequence consists of seven static PPI network snapshots: at the first snapshot, network have 70% high confidence interactions of original network. at second snapshot, we add 5% high confidence interactions. Now the network have 75% interactions of original which have greater confidence value than the rest interactions. We continue to add 5% interaction until the network has hole 100% interaction of original network. Then we get a real-world dynamic network with seven static PPI network snapshots. Then we generate two randomized versions of network, as follow:

**Randomizes only temporal aspect of network**: Since the difference between dynamic NA and static NA is that the former accounts for the temporal aspect of the data more explicitly than the latter, we first randomizes only temporal aspect of network, which means randomized network will preserve as much structure as possible of dynamic network. They are only different from each other in time information. This way, the only difference observed between Twadn’s and NetCoffee2’s performance will be the consequence of considering the temporal aspect of the data. We randomize network with a parameter the noisy level *p*, the larger the *p* value, the more noise is added. Given the noise level *p* and *G*=(*g*_1_,*g*_2_,...,*g*_7_),*g*_*i*_=(*V*_*i*_,*E*_*i*_), for each *e*_*ab*_(*a*∈*V*_*i*_,*b*∈*V*_*i*_) in *E*_*i*_, with probability *p*, we arbitrarily select *e*_*cd*_(*c*∈*V*_*j*_,*d*∈*V*_*j*_) from another snapshot *g*_*j*_(*j*≠*i*) and swap this two interactions. The specific method of swap is to delete the edge *e*_*ab*_ in the snapshot *g*_*i*_ and add an edge *e*_*cd*_. At the same time, delete *e*_*cd*_ in the snapshot *g*_*j*_ and increase *e*_*ab*_. By randomizing only temporal aspect of network, an dynamic network can be aggregated into the same static network as its noisy version’s.

**Randomizes both temporal aspect and structure aspect of network**: To observe the performance of Twadn and NetCoffee2 in different sets of noisy versions of the original network using a somewhat more flexible randomization scheme that does not conserve the structure of the flattened version of the original dynamic network, we randomize both temporal aspect and structure aspect of network. Given the noisy level *p* and *G*=(*g*_1_,*g*_2_,...,*g*_7_),*g*_*i*_=(*V*_*i*_,*E*_*i*_), for each *e*_*ab*_(*a*∈*V*_*i*_,*b*∈*V*_*i*_) in *E*_*i*_, with probability *p*, we arbitrarily select *e*_*cd*_(*c*∈*V*_*j*_,*d*∈*V*_*j*_) from another snapshot *g*_*j*_(*j*≠*i*), if there is no edge connection between node *a* and *d*, and there is no edge connection between node *b* and node *c*, then connect *a* and *d*, *b* and *c*. If the process of adding noise creates a loop (i.e., an edge from a node to itself) or a multiple link (i.e., duplicate edge between the same nodes), then we undo it and re-randomly select another edge to do the above process. By randomizing both temporal aspect and structure aspect of network, the resulting compressed version of the static network is different from the original dynamic network.

#### Evaluation measures

For NA of Drosophila dynamic networks with increased noise, we know the true alignment results (the same protein can be considered a homologous protein). So we use the Alignment Score, which is the algorithm’s objective function value (Eq. 3) and node correctness (NC) to measure the network alignment results and the NC can be calculate as follow:

9 $$ NC=\frac{N_{correct}}{N_{all}}  $$

where *N*_*correct*_ represents the number of correctly aligned protein pairs and *N*_*all*_ represents the count of all protein pairs. The greater the node-correctness is, the better the algorithm is. When noise is added to the dynamic network only on the timing information, the dynamic network will become more and more dissimilar to the original network as the level of noise increases, while the dynamic network will be compressed into the same static network. Then as the noise level increases, the comparison result of Twadn will become worse and worse, and the result of NetCoffee2 should not change much. When randomizes both temporal aspect and structure aspect of network, if the dynamic network is compressed to a static version, different static networks will be obtained. As noise level increase, the noise and original versions of dynamic and static networks become more and more dissimilar. Then if Twadn and NetCoffee2 are used to compare the dynamic network with the static network respectively, the comparison results of the two algorithms should be worse as the noise level increases.

#### Result

**Fig. 5:**
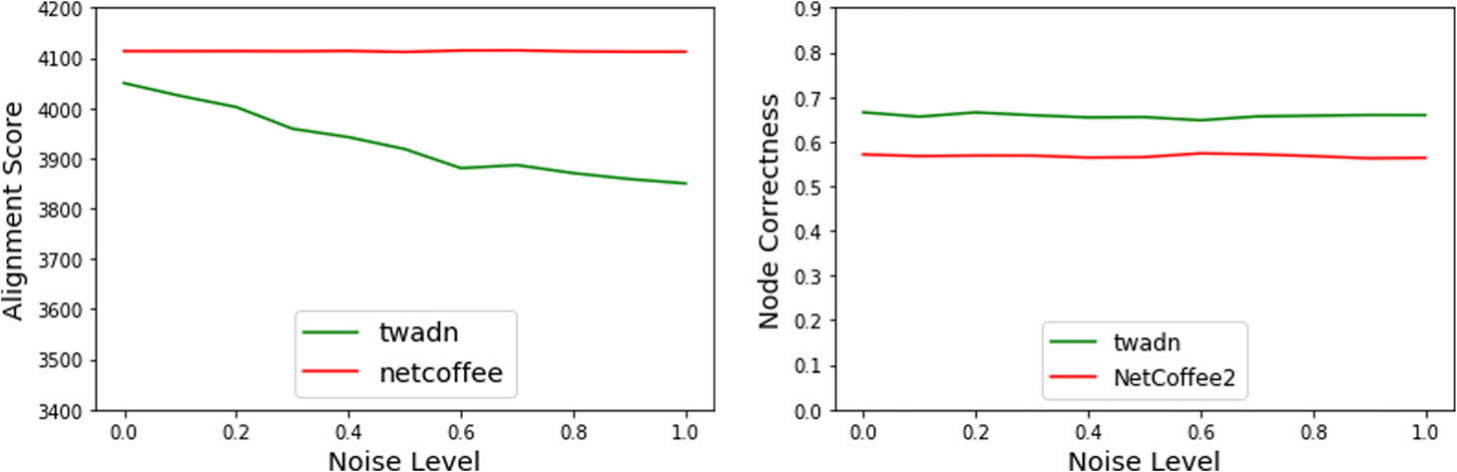
The performance of node correctness and alignment score when randomizing only temporal aspect of network

**Fig. 6:**
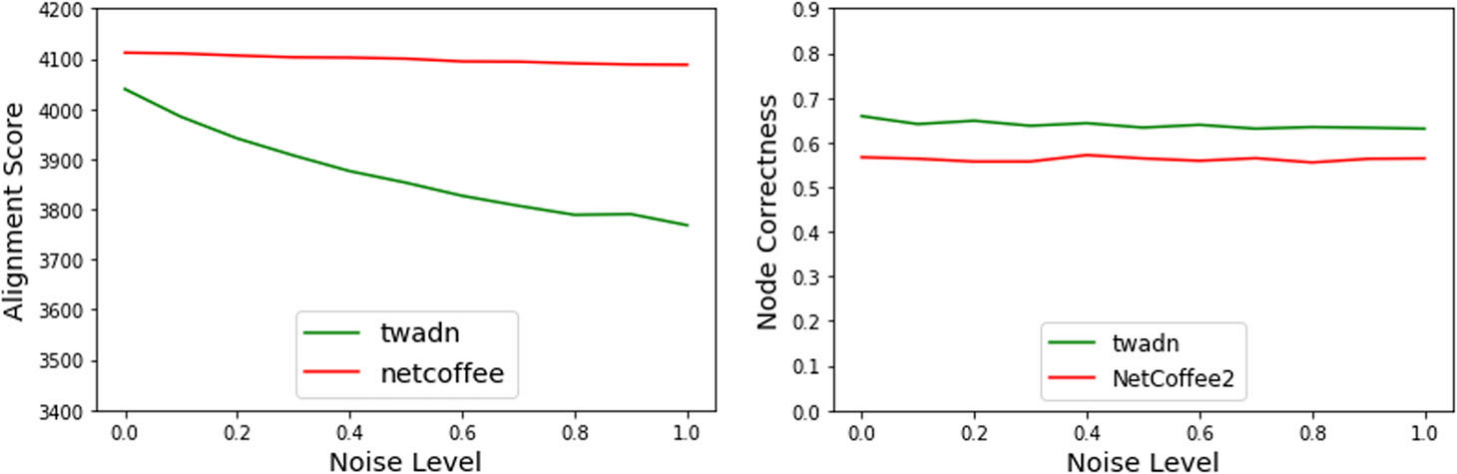
The performance of node correctness and alignment score when randomizing both temporal aspect and structure aspect of network

As Fig. 5 shows, the alignment score of Twadn decrease with the increase of noisy, while the alignment score of NetCoffee2 does not change when randomizing only temporal aspect. It is reasonable since the flattened version of noisy of dynamic network is the same as original network’s. The alignment quality of NetCoffee2 and Twadn both decrease when randomizing both temporal aspect and structure aspect of network in Fig. 6. This illustrates that Twadn really capture the time information of dynamic network compared with NetCoffee2. In other hand, the node-correctness of Twadn is higher than NetCoffee2 in every noisy level, which means Twadn is superior to NetCoffee2. We think it is reasonable as Twadn capture the time information of dynamic network while NetCoffee2 does not.

## Discussion

Although the experimental results have been able to achieve better results than existing algorithms. There are still many problems in the work done in this paper that can be further explored: 1) The algorithm temporarily does not support multiple dynamic networks for simultaneous alignment. However, according to the framework of the simulated annealing algorithm, it is hoped that multiple networks can be aligned at the same time in the future. 2) The algorithm is currently only used in PPI networks, and it is expected to be applied to other networks in the future, such as gene regulatory networks, metabolic networks, etc.

## Conclusion

NA is a very important computational framework for understanding molecular function and phylogenetic relationships. Although many NA methods have been developed in the last decade, most of these focused on aligning proteins in static PPI networks. All species and PPIs evolve in different speed. Therefore, there is an urgent demand to develop efficient computational tools to deal with these dynamic networks. To supplement this shortcoming, a novel method Twadn based on the dynamic model of networks was proposed, which can include the time information of molecular interactions. We construct a 5-tupe-feature vector and an optimal warping path to extract topology structures and evolving patterns of all nodes in networks. Twadn was applied in both synthetic datasets and real biological datasets. The synthetic dataset was generated based on a scale-free gene duplication model. Results show that Twadn is superior to DynaMAGNA++ and DynaWAVE in synthetic network. At the same time, in order to show that the Twadn can capture timing information compared to the static NA, we add timing information and noise to the Drosophila protein interaction network, and then run the Twadn and NetCoffee2, experimental results in line with our expectations. Dynamic NA algorithm do capture timing information. It suggests that Twadn is a versatile and efficient alignment tool that can be applied to dynamic network. Hopefully, its application can benefit the research community in the fields of molecular function and evolution.

## Data Availability

The source code of Twadn is freely available at: https://github.com/screamer/twadn.

## Abbreviations

AUPR: Area under the precision-recall curve
AUROC: Area under the receiver operating characteristic curve
DN: Dynamic network
DTW: Dynamic time warping
FPR: False positive rate
NA: Network alignment
NC: Node correctness
PPI: Protein-protein interaction
ROC: Receiver operating characteristic
TPR: True positive rate
Twadn: Time warping based alignment algorithm for pairwise dynamic networks
